# EGFR-Targeted Granzyme B Expressed in NK Cells Enhances Natural Cytotoxicity and Mediates Specific Killing of Tumor Cells

**DOI:** 10.1371/journal.pone.0061267

**Published:** 2013-04-03

**Authors:** Pranav Oberoi, Robert A. Jabulowsky, Hayat Bähr-Mahmud, Winfried S. Wels

**Affiliations:** Chemotherapeutisches Forschungsinstitut Georg-Speyer-Haus, Frankfurt am Main, Germany; Karolinska Institutet, Sweden

## Abstract

Natural killer (NK) cells are highly specialized effectors of the innate immune system that hold promise for adoptive cancer immunotherapy. Their cell killing activity is primarily mediated by the pro-apoptotic serine protease granzyme B (GrB), which enters targets cells with the help of the pore-forming protein perforin. We investigated expression of a chimeric GrB fusion protein in NK cells as a means to augment their antitumoral activity. For selective targeting to tumor cells, we fused the epidermal growth factor receptor (EGFR) peptide ligand transforming growth factor α (TGFα) to human pre-pro-GrB. Established human NKL natural killer cells transduced with a lentiviral vector expressed this GrB-TGFα (GrB-T) molecule in amounts comparable to endogenous wildtype GrB. Activation of the genetically modified NK cells by cognate target cells resulted in the release of GrB-T together with endogenous granzymes and perforin, which augmented the effector cells' natural cytotoxicity against NK-sensitive tumor cells. Likewise, GrB-T was released into the extracellular space upon induction of degranulation with PMA and ionomycin. Secreted GrB-T fusion protein displayed specific binding to EGFR-overexpressing tumor cells, enzymatic activity, and selective target cell killing in the presence of an endosomolytic activity. Our data demonstrate that ectopic expression of a targeted GrB fusion protein in NK cells is feasible and can enhance antitumoral activity of the effector cells.

## Introduction

Natural killer (NK) cells are highly specialized effectors of the innate immune system. They play an important role in the defense against viral infection and the elimination of neoplastic cells [1]. Natural cytotoxicity of NK cells can be triggered rapidly upon appropriate stimulation, and is regulated by a complex balance of signals from germline-encoded activating and inhibitory cell surface receptors [2]. Following target cell recognition and activation, lytic granules within the effector cells are polarized towards the immunological synapse, where they fuse with the plasma membrane and release their contents into the synaptic cleft between effector and target cell [3], [4]. Similar to cytotoxic T cells, cell killing by NK cells is primarily mediated by the granzyme family of serine proteases, and the pore-forming protein perforin [5]. Thereby the pro-apoptotic granzyme B (GrB) plays the most crucial role for cytotoxicity [6]. Initially, GrB is expressed as an inactive precursor protein. This pre-pro-GrB carries an N-terminal signal peptide, directing packaging of the protein into secretory granules, followed by the activation dipeptide Gly-Glu. Removal of this peptide by the cysteine protease cathepsin C generates the enzymatically active form of GrB [7], which is stored together with other granzymes and perforin in the dense core of lytic granules. Upon release from cytotoxic lymphocytes, GrB enters target cells in cooperation with perforin, and rapidly induces apoptosis via caspase-dependent and caspase-independent mechanisms [8].

Owing to the relatively small size of 227 amino acid residues for mature GrB, its broad substrate specificity, and its ability to bypass common apoptosis resistance mechanisms in tumor cells, GrB has been employed as an effector molecule for the generation of recombinant cell death-inducing fusion proteins [9], [10]. Since GrB is of human origin, such immunotoxin-like molecules are expected to circumvent immunogenicity and other complications frequently associated with recombinant toxins of plant or bacterial origin [11]. Recombinant GrB and chimeric GrB fusion proteins that harbor peptide ligands or antibody domains for tumor-specific cell recognition have been successfully produced in bacterial, yeast and mammalian expression systems [12], [13], [14], [15], [16], [17], and have been shown to retain potent cytotoxicity upon targeted delivery into tumor cells [12], [13], [15], [18], [19], [20].

Here, we investigated feasibility and consequences of expression of a chimeric GrB fusion protein in human NK cells, utilizing established NKL cells as a model. NK cells possess all pathways required for processing, packaging, and triggered release of endogenous wildtype GrB, which may be readily employed by an ectopically expressed retargeted GrB derivative. For selective targeting to tumor cells, we fused the epidermal growth factor receptor (EGFR) peptide ligand transforming growth factor α (TGFα) to human pre-pro-GrB. EGFR overexpression and aberrant activation have been found in many tumors of epithelial origin, and have been shown to contribute to malignant transformation [21]. Due to its accessibility from the extracellular space, EGFR constitutes an attractive target for therapeutic antibodies and cytotoxic antibody or growth factor fusion proteins [15], [22], [23], [24]. NK cells transduced with a lentiviral vector encoding the GrB-TGFα fusion protein expressed the chimeric GrB-T molecule in amounts comparable to endogenous wildtype GrB, which augmented natural cytotoxicity of the genetically modified NK cells against NK-sensitive targets. Furthermore, induction of degranulation resulted in the release of GrB-T from vesicular compartments into the extracellular space. The secreted fusion protein was functionally active, and displayed specific binding to EGFR-overexpressing tumor cells as well as selective target cell killing in the presence of an endosomolytic activity.

## Results

### Expression of the granzyme B-TGFα fusion protein GrB-T in NK cells

**Figure 1:**
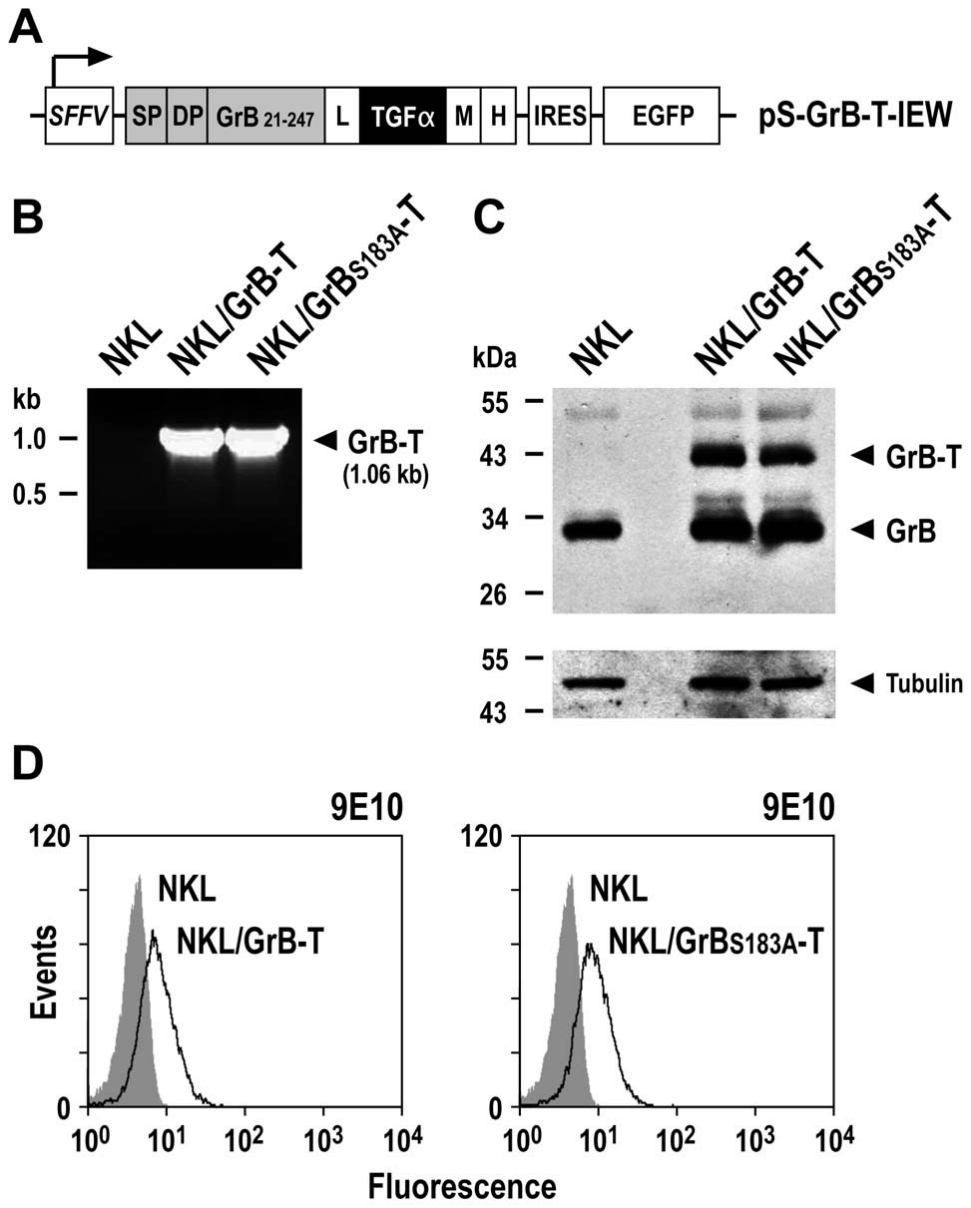
Expression of the granzyme B-TGFα fusion protein GrB-T in NK cells. (A) Schematic representation of the lentiviral transfer vector pS-GrB-T-IEW that encodes under the control of the Spleen Focus Forming Virus promoter (SFFV) a fusion of human GrB with TGFα, followed by an internal ribosome entry site (IRES) and cDNA encoding enhanced green fluorescent protein (EGFP) as a marker. SP, GrB signal peptide; DP, GrB activation dipeptide; GrB_21–247_, mature form of GrB; L, flexible linker; M, Myc tag; H, hexa-histidine tag. The similar transfer vector pS-GrB_S183A_-T-IEW encodes enzymatically inactive mutant GrB_S183A_ fused to TGFα (not shown). After transduction with S-GrB-T-IEW or S-GrB_S183A_-T-IEW vector particles, EGFP-expressing NKL/GrB-T and NKL/GrB_S183A_-T cells were enriched by flow cytometric cell sorting, and analyzed for GrB-T expression. (B) GrB-T mRNA expression in NKL/GrB-T and NKL/GrB_S183A_-T cells was verified by semi-quantitative RT-PCR. (C) Expression of GrB-T proteins in NKL/GrB-T and NKL/GrB_S183A_-T cells was investigated by immunoblot analysis of cell lysates with GrB-specific antibody. γ-Tubulin was analyzed as a loading control. (D) Expression of GrB-T proteins in NKL/GrB-T and NKL/GrB_S183A_-T cells was confirmed by intracellular staining with Myc-tag-specific antibody and flow cytometry (open areas). In all experiments parental NKL cells served as controls.

cDNA encoding human pre-pro-GrB was fused via a flexible (Gly_4_Ser)_4_-His_6_ linker to a sequence encoding the EGFR-specific peptide ligand TGFα followed by C-terminal Myc and hexa-histidine tags in the lentiviral transfer vector pSIEW, that also encodes enhanced green fluorescent protein (EGFP) as a marker (Fig. 1A). After generation of lentiviral particles and transduction of human NKL cells, EGFP-expressing NKL/GrB-T cells were enriched by two rounds of flow cytometric cell sorting. Likewise, NKL/GrB_S183A_-T control cells were generated by lentiviral transduction with a vector encoding a fusion of TGFα with the enzymatically inactive GrB mutant GrB_S183A_ [14]. In the enriched NKL/GrB-T and NKL/GrB_S183A_-T cell populations, GrB-T and GrB_S183A_-T mRNA expression was verified by semi-quantitative RT-PCR analysis (Fig. 1B). GrB-T and GrB_S183A_-T proteins with an apparent molecular mass of 40 kDa were readily detected in addition to endogenous wildtype GrB by immunoblot analysis of NKL cell lysates with GrB-specific antibody (Fig. 1C), confirming expression and integrity of the fusion proteins. Intracellular staining of NKL/GrB-T and NKL/GrB_S183A_-T cells with Myc-tag-specific antibody also verified expression of GrB-T and GrB_S183A_-T in transduced NKL cells (Fig. 1D).

### Ectopic expression of GrB-T enhances natural cytotoxicity of NKL cells

**Figure 2:**
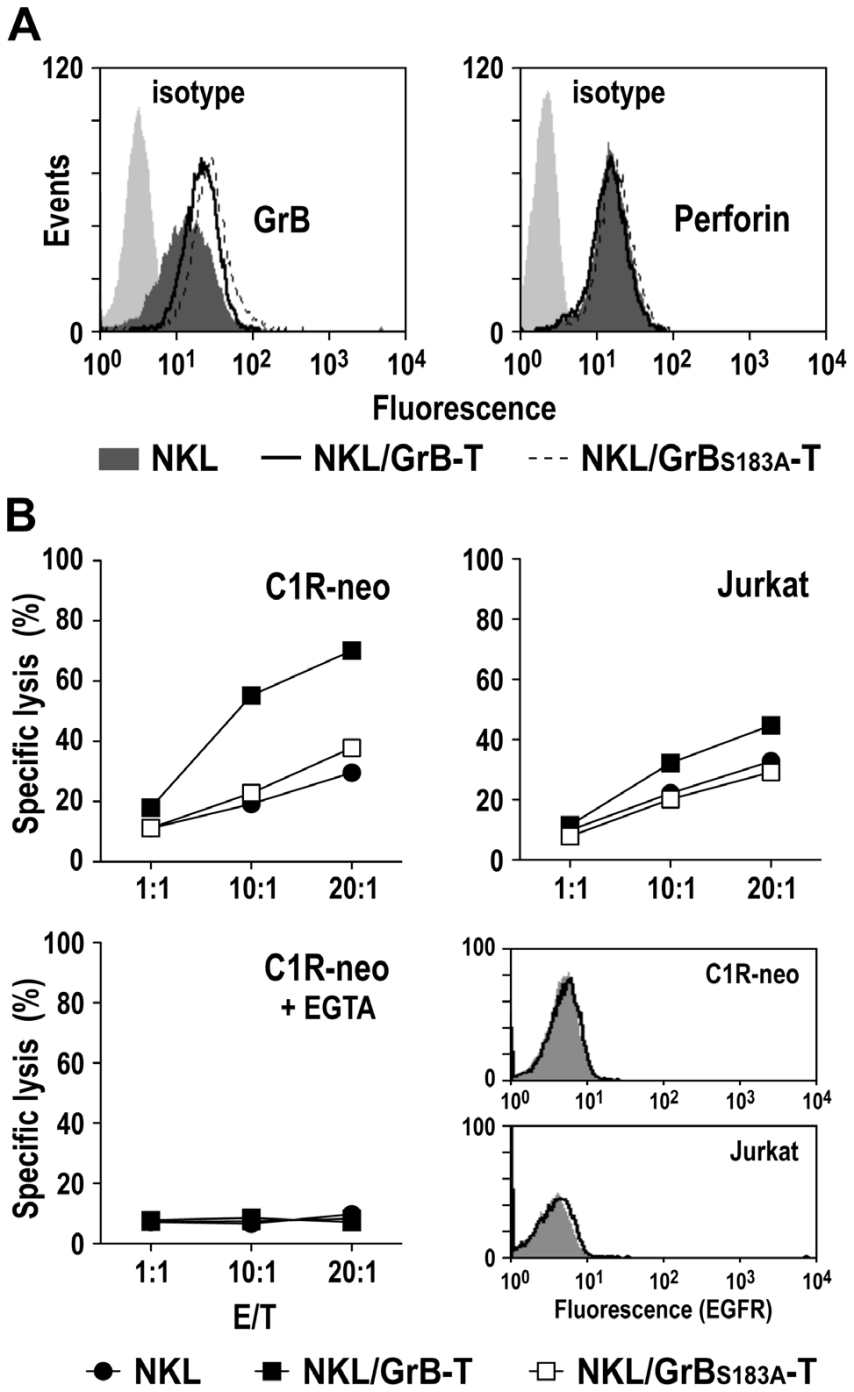
Natural cytotoxicity of NKL/GrB-T and NKL/GrB_S183A_-T cells. (A) Total levels of GrB and perforin expressed by parental NKL (dark gray areas), NKL/GrB-T (bold lines) and NKL/GrB_S183A_-T cells (dotted lines) was analyzed by intracellular staining with GrB-specific antibody (left) or perforin-specific antibody (right) and flow cytometry. NKL cells incubated with isotype-matched antibodies served as controls (light gray areas). (B) Cytotoxicity of NKL/GrB-T (filled squares) and NKL/GrB_S183A_-T cells (open squares) towards C1R-neo and Jurkat cells was determined in FACS-based cytotoxicity assays at different effector to target ratios (E/T). Parental NKL cells (filled circles) were included for comparison. Dependence of target cell killing on the release of granular proteins was confirmed by incubating C1R-neo target cells with NKL effector cells in the presence of 2 mM of the Ca^2+^ chelator EGTA. Representative data of one of three independent experiments are shown. Absence of EGFR expression on the surface of C1R-neo and Jurkat cells was confirmed by flow cytometry with EGFR-specific antibody (open areas). Cells treated only with secondary antibody served as controls (shaded areas).

Total levels of GrB in parental and genetically modified NKL cells were analyzed by intracellular staining. We observed a marked increase in total GrB in NKL/GrB-T and NKL/GrB_S183A_-T cells in comparison to unmodified NKL, attributed to GrB-T and GrB_S183A_-T fusion proteins ectopically expressed in addition to endogenous wildtype GrB (Fig. 2A, left panel). In contrast, expression levels of perforin remained unchanged by transduction with the lentiviral vectors, and were comparable in NKL, NKL/GrB-T and NKL/GrB_S183A_-T cells (Fig. 2A, right panel).

Next, we investigated natural cytotoxicity of NKL/GrB-T and NKL/GrB_S183A_-T cells in FACS-based assays with NKL-sensitive C1R-neo and Jurkat cells as targets. Parental NKL cells were included for comparison. As expected, co-incubation of Jurkat and C1R-neo cells with unmodified NKL cells for 4 h revealed significant cell killing activity at different E/T ratios, which was very similar for NKL/GrB_S183A_-T cells that express the enzymatically inactive fusion protein (Fig. 2B, upper panels). In contrast, natural cytotoxicity of NKL/GrB-T cells was markedly increased, resulting in 70% lysis of C1R-neo cells at an E/T ratio of 20∶1, while cell killing by NKL/GrB_S183A_-T and parental NKL was 38 and 30% under these conditions. Also cytotoxicity of NKL/GrB-T cells towards Jurkat cells was enhanced, albeit to a lower extent (45% cell killing at an E/T ratio of 20∶1 versus 29 and 33% cell killing for NKL/GrB_S183A_-T and NKL, respectively).

Release of secretory granular proteins and perforin activity require the presence of Ca^2+^. When cell killing experiments with C1R-neo target cells were performed in the presence of the Ca^2+^ chelator EGTA, cytotoxicity of NKL/GrB-T, NKL/GrB_S183A_-T, and NKL cells was abolished, indicating dependence of cell killing on granule exocytosis and perforin (Fig. 2B, lower panel, left). C1R-neo and Jurkat cells do not express EGFR (Fig. 2B, lower panel, right). This suggests that in the absence of the TGFα target receptor, GrB-T fusion protein can act in a manner similar to endogenous wildtype GrB and enhance natural cytotoxicity of NKL cells upon encounter of NK-sensitive targets. For this increased target cell killing, enzymatic activity of the GrB-T fusion protein as well as intact granule exocytosis and perforin activity were required.

### Cytotoxicity of NKL/GrB-T cells towards EGFR-expressing tumor cells

**Figure 3:**
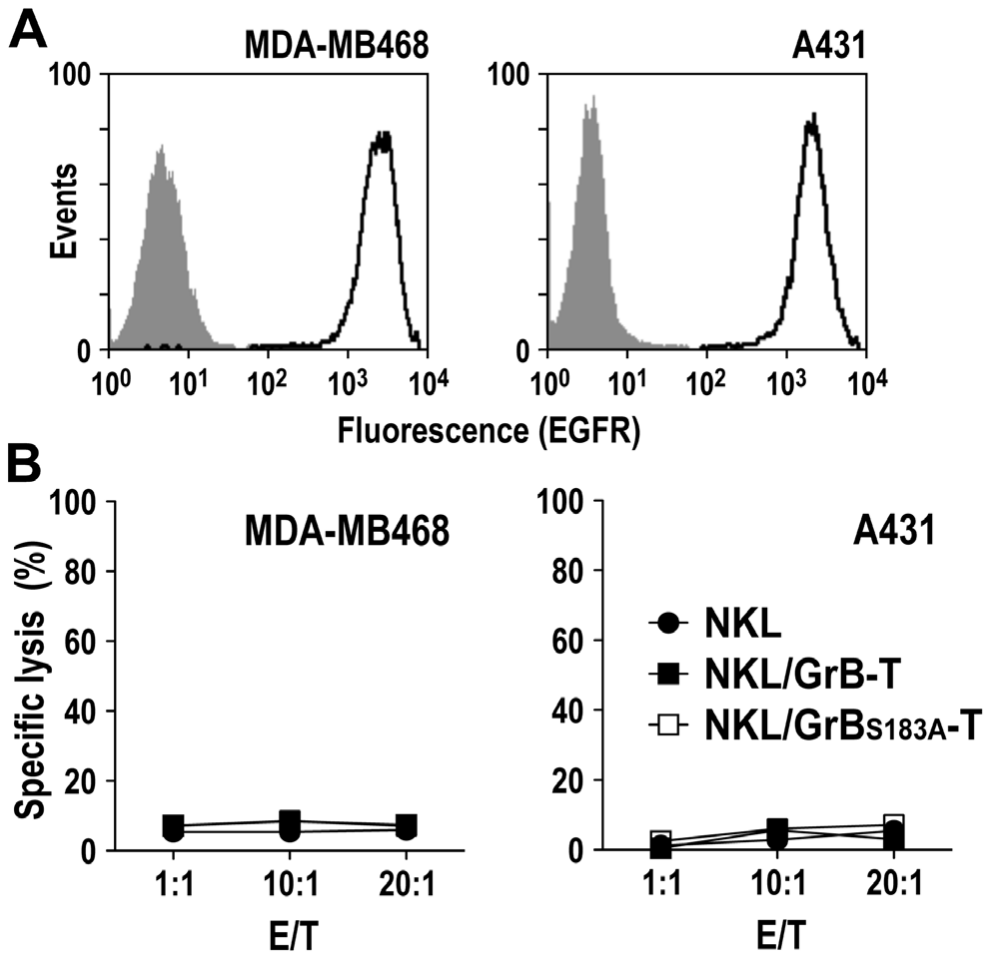
Cytotoxicity of NKL/GrB-T and NKL/GrB_S183A_-T cells towards EGFR-expressing tumor cells. (A) Expression of EGFR on the surface of MDA-MB468 breast carcinoma and A431 squamous cell carcinoma cells was determined by flow cytometry with EGFR-specific antibody (open areas). Cells treated only with secondary antibody served as controls (shaded areas). (B) Cytotoxicity of NKL/GrB-T (filled squares) and NKL/GrB_S183A_-T cells (open squares) towards MDA-MB468 and A431 cells was determined in FACS-based cytotoxicity assays at different E/T ratios. Parental NKL cells (filled circles) were included for comparison.

Next, we investigated whether NKL/GrB-T cells also display enhanced cytotoxic activity against EGFR-expressing tumor cells employing established human MDA-MB468 breast carcinoma and A431 squamous cell carcinoma cells as targets. These cells express high levels of EGFR on the cell surface as confirmed by flow cytometry (Fig. 3A). Cell killing activity of NKL/GrB-T and NKL/GrB_S183A_-T cells against MDA-MB468 and A431 cells was assessed in FACS-based assays upon co-incubation of effector and target cells for 4 h at different ratios. Parental NKL cells were included for comparison (Fig. 3B). In contrast to Jurkat and C1R-neo cells, at the tested E/T ratios EGFR-expressing MDA-MB468 and A431 cells were resistant to lysis by parental NKL cells as well as the genetically modified derivatives NKL/GrB-T and NKL/GrB_S183A_-T.

**Figure 4:**
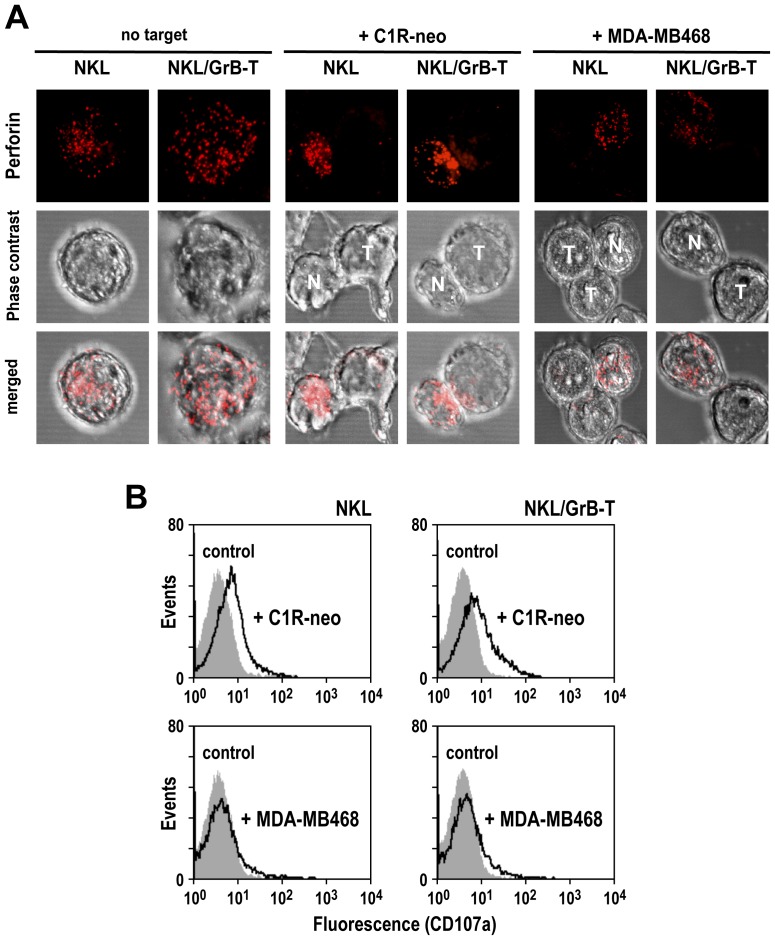
Conjugate formation and redistribution of cytotoxic granules upon target cell contact. (A) Parental NKL and NKL/GrB-T cells were incubated with C1R-neo or MDA-MB468 target cells at an E/T ratio of 1∶1 for 1 h at 37°C, stained with perforin-specific antibody as indicated (red), and analyzed by confocal laser scanning microscopy. As a control, NK cells were incubated without target cells. Maximum projection of a z-stack series is shown in each case. N: NK cell; T: target cell. (B) To assess activation of NK cells upon target cell contact, parental NKL and NKL/GrB-T cells were incubated with C1R-neo (upper panel) or MDA-MB468 target cells (lower panel) at an E/T ratio of 1∶1 for 5 h at 37°C, before analysis of cell surface-associated degranulation marker CD107a by flow cytometry (open areas). NKL cells incubated in the absence of target cells served as controls (shaded areas).

NK cell-mediated cytotoxicity requires efficient activation of the effector cells upon recognition of target cells. To investigate polarization of cytotoxic granules to the site of contact and release of granular contents into the synaptic cleft, we performed confocal laser scanning microscopy analysis with mixed cultures of NKL, and NKL-sensitive or NKL-resistant cancer cells as targets. Parental NKL or NKL/GrB-T cells were added to C1R-neo or MDA-MB468 cells, and stained with perforin-specific antibody to visualize cytotoxic granules and distinguish effector and target cells. NK cells in the absence of targets were included for comparison. In NK cells incubated for 1 h with C1R-neo, perforin-containing granules were concentrated at the interface with the target cells (Fig. 4A, middle panel), indicative of effector cell activation. In contrast, upon incubation with MDA-MB468, the majority of NKL and NKL/GrB-T cells formed conjugates with the targets, but showed little or no polarization of cytotoxic granules towards the site of contact (Fig. 4A, right panel). This resembled the distribution pattern observed in unstimulated NKL cultured without target cells (Fig. 4A, left panel). To assess differences in effector cell activation by the cancer cells, we subsequently analyzed activation-induced degranulation of NKL and NKL/GrB-T cells upon contact with C1R-neo and MDA-MB468. Cells were co-incubated at an E/T ratio of 1∶1 for 5 h, before surface expression of the lysosomal associated membrane protein LAMP-1 (CD107a) was measured by flow cytometry. This functional marker for CTL and NK cell activity resides in the membrane of lytic granules, and is mobilized to the cell surface following activation-induced granule exocytosis. While CD107a levels were markedly increased on the surface of NKL and NKL/GrB-T cells upon encounter of C1R-neo cells (Fig. 4B, upper panel), only marginal changes in CD107a expression were observed upon contact of the effector cells with MDA-MB468 target cells (Fig. 4B, lower panel).

Hence, while NKL and NKL/GrB-T cells formed conjugates with the EGFR-expressing targets, this interaction did not induce effector cell activation, degranulation and target cell killing.

### Release of GrB-T protein from NKL/GrB-T cells upon activation-induced degranulation

**Figure 5:**
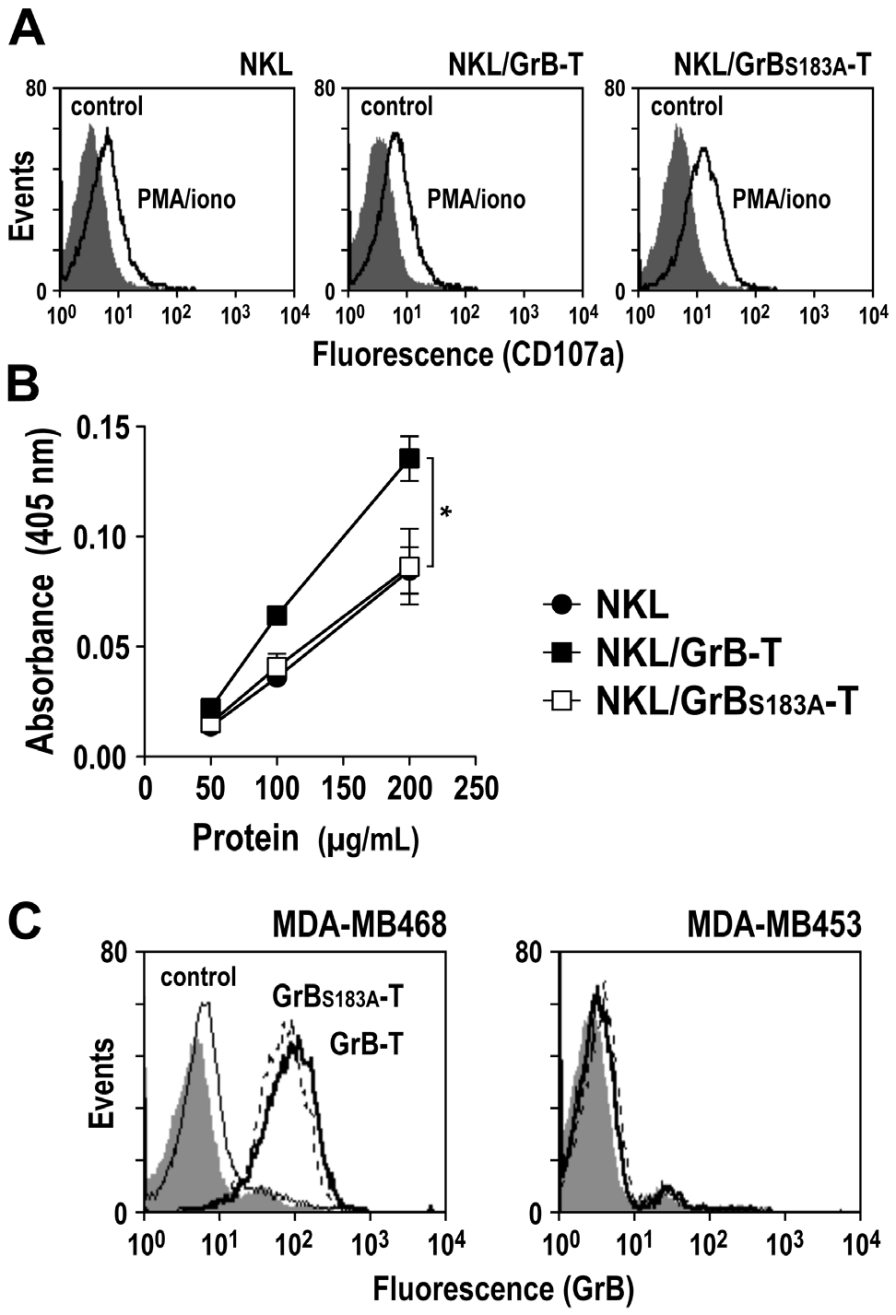
Release of GrB-T protein upon degranulation of NK cells. (A) Degranulation of NKL, NKL/GrB-T and NKL/GrB_S183A_-T cells was induced by treatment with PMA and ionomycin for 5 h at 37°C, and culture supernatants were harvested. To confirm activation of cells, CD107a expression was analyzed by flow cytometry (open areas). Unstimulated cells served as controls (shaded areas). (B) To determine enzymatic activity of GrB and GrB-T proteins, GrB-specific peptide substrate Ac-IETD-pNA was incubated with 50 to 200 µg/mL of total proteins from supernatants of activated NKL (filled circles), NKL/GrB-T (filled squares) or NKL/GrB_S183A_-T cells (open squares). Substrate cleavage was determined by measuring the absorbance at 405 nm. Mean values ± SEM are shown; n = 4. *, *P*<0.05. (C) Binding of GrB-T (bold line) and mutant GrB_S183A_-T protein (dotted line) released by activated NKL/GrB-T and NKL/GrB_S183A_-T cells to EGFR-positive MDA-MB468 and EGFR-negative MDA-MB453 breast carcinoma cells was determined by flow cytometry with GrB-specific antibody. Cells treated with medium (shaded areas) or proteins released by activated parental NKL cells (regular line) served as controls.

To facilitate release of GrB-T fusion protein from NKL cells for functional analysis, NKL/GrB-T and NKL/GrB_S183A_-T cells were stimulated with PMA and ionomycin, and induction of degranulation was confirmed by demonstrating enhanced expression of CD107a on the cell surface (Fig. 5A). Enzymatic activity of released proteins was analyzed using the GrB-specific peptide substrate Ac-IETD-pNA (Fig. 5B). Thereby concentration-dependent GrB activity was found in supernatant of activated parental NKL cells. As expected, this was very similar for NKL/GrB_S183A_-T cells that express enzymatically inactive fusion protein but also harbor endogenous wildtype GrB. In contrast, markedly enhanced GrB activity was detected in supernatant from activated NKL/GrB-T, indicating release of enzymatically active GrB-T fusion protein from these cells in addition to endogenous wildtype GrB.

Functionality of the TGFα domain of GrB-T was investigated in cell binding experiments with EGFR-overexpressing MDA-MB468 cells. As a control, human MDA-MB453 breast carcinoma cells were included, which are negative for EGFR [20]. Tumor cells were incubated with culture supernatants of activated NKL/GrB-T and NKL/GrB_S183A_-T cells, and surface-bound proteins were detected by flow cytometry with fluorochrome-labeled GrB-specific antibody. For proteins from NKL/GrB-T and NKL/GrB_S183A_-T cells, strong binding to the surface of MDA-MB468 cells, but not EGFR-negative MDA-MB453 cells was found (Fig. 5C). Endogenous GrB released from parental NKL cells displayed only marginal binding to MDA-MB468 and MDA-MB453 cells.

These data show that GrB-T and GrB_S183A_-T fusion proteins were indeed released from NKL cells upon activation-induced degranulation. While GrB-T was bifunctional and displayed enzymatic activity as well as EGFR-specific binding, the GrB_S183A_-T control protein retained cell binding, but as expected did not cleave GrB substrate.

### Cytotoxic activity of GrB-T protein from activated NKL cells

**Figure 6:**
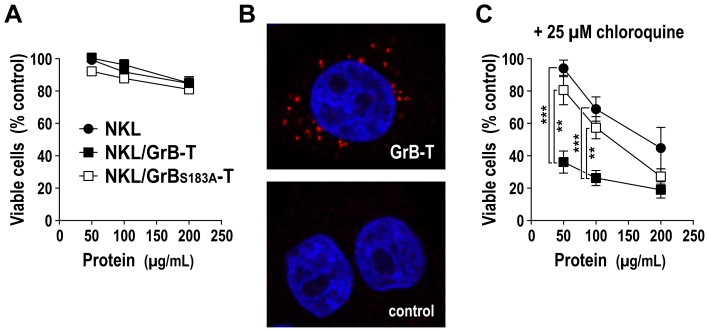
Cytotoxic activity of proteins released by activated NK cells. (A) EGFR-positive MDA-MB468 cells were treated with increasing concentrations of total proteins from supernatants of activated NKL (closed circles), NKL/GrB-T (closed squares), or NKL/GrB_S183A_-T cells (open squares). After 24 h, the relative number of viable cells in comparison to medium-treated controls was determined in WST-1 assays. Mean values ± SEM are shown; n = 9. (B) To investigate uptake and intracellular localization of GrB-T protein, MDA-MB468 cells were treated with GrB-T-containing supernatant from NKL/GrB-T cells for 60 min at 4°C, washed and incubated for another 90 min at 37°C, before staining with GrB-specific antibody (red) and confocal laser scanning microscopy. Control cells were incubated with supernatant from activated parental NKL cells. Nuclei were stained with DAPI (blue). Merged images are shown. (C) To facilitate release of internalized proteins from endosomes, MDA-MB468 were treated with total proteins from supernatants of activated NKL, NKL/GrB-T, or NKL/GrB_S183A_-T cells in the presence of 25 µM of the endosomolytic reagent chloroquine, and the relative number of viable cells in comparison to controls treated with chloroquine-containing medium was determined in WST-1 assays. Mean values ± SEM are shown; n = 9. ***, *P*<0.001; **, *P*<0.01.

To analyze cytotoxic activity of GrB-T protein from NKL/GrB-T cells, the cells were treated with PMA and ionomycin as described above, cell-free supernatant was collected, and increasing concentrations from 50 to 200 µg/mL of total granular proteins were added to MDA-MB468 cells. Equal concentrations of proteins released by NKL/GrB_S183A_-T and parental NKL cells were included as controls. After 24 h, the relative number of viable cells in comparison to target cells cultured in PMA- and ionomycin-containing medium in the absence of NK cell proteins was determined in WST-1 metabolization assays (Fig. 6A). A slight reduction in cell viability was observed at high protein concentrations. However, this was independent from the source of the granular proteins, and most likely due to the activity of endogenous granzymes and perforin released by the different NKL cell derivatives. To test whether insufficient intracellular uptake of GrB-T protein was responsible for the lack of specific cell killing, MDA-MB468 cells were treated with supernatant from activated NKL/GrB-T cells, and uptake of GrB-T after 1.5 h at 37°C was analyzed by confocal laser scanning microscopy using fluorochrome-conjugated GrB antibody. In the majority of target cells, punctuate staining within the cytoplasm was found (Fig. 6B, upper panel), suggesting receptor-mediated endocytosis of GrB-T upon binding to EGFR and routing to endosome-like vesicular structures. MDA-MB468 cells incubated with the same amount of protein from activated parental NKL cells containing endogenous wildtype GrB did not display intracellular staining with GrB-specific antibody (Fig. 6B, lower panel).

To test whether release from endosomal vesicles in target cells could facilitate access of GrB-T to cytosolic GrB substrates and enable cytotoxic activity, next we treated target cells with increasing concentrations of total granular proteins from activated NKL/GrB-T cells in the presence of chloroquine. Indeed, addition of the endosomolytic reagent strongly enhanced cytotoxicity of GrB-T protein, resulting in effective and concentration-dependent killing of target cells (Fig. 6C). Interestingly, chloroquine also increased cytotoxic activity of endogenous granular proteins from NKL and NKL/GrB_S183A_-T cells. However, in these cases much higher protein concentrations were required to achieve significant cell killing.

### Specificity of GrB-T-mediated cell killing

**Figure 7:**
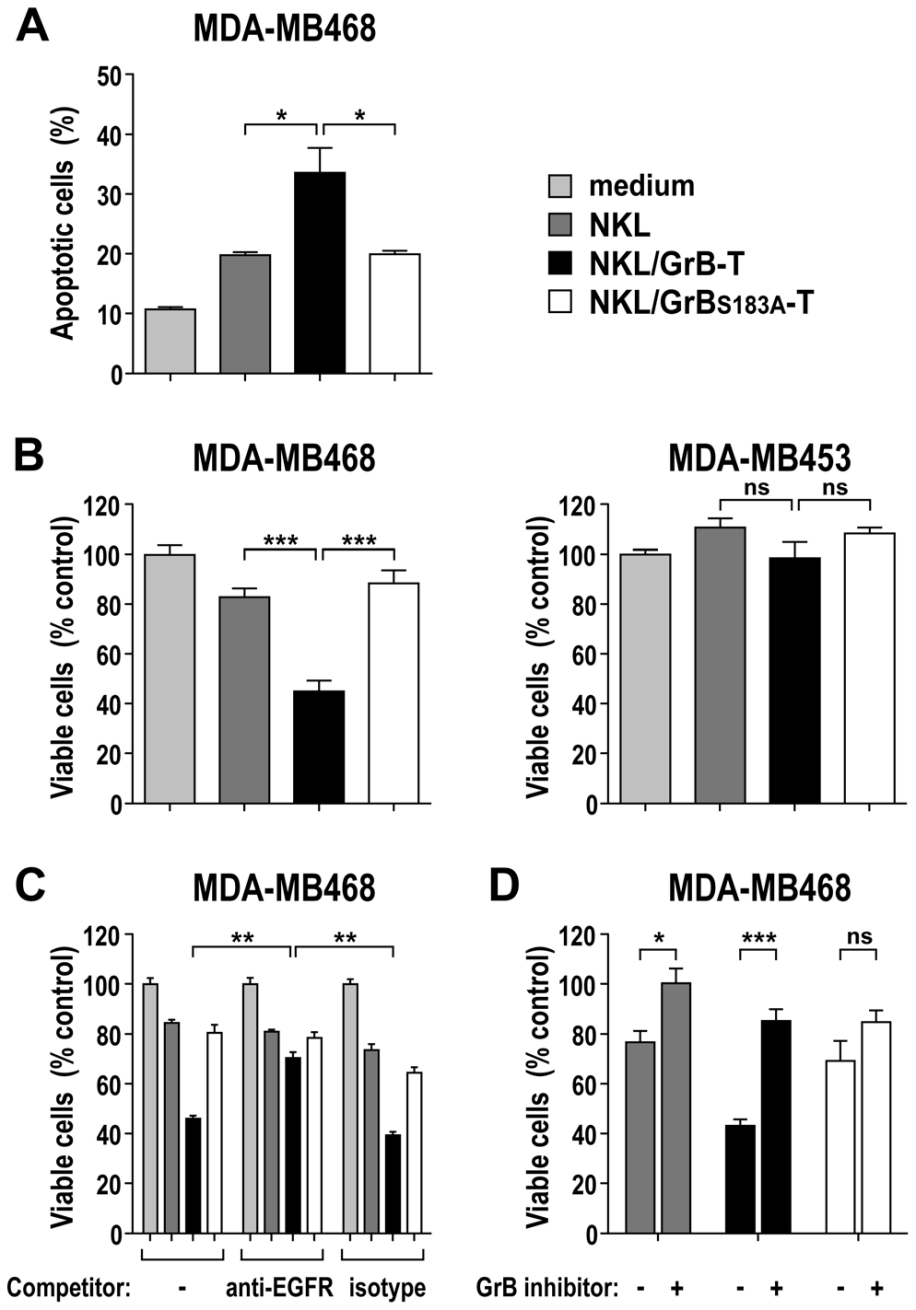
Selective cytotoxicity of GrB-T fusion protein. (A) Induction of apoptosis after treatment of MDA-MB468 cells for 24 h with 100 µg/mL of total proteins from supernatants of activated NKL, NKL/GrB-T, or NKL/GrB_S183A_-T cells in the presence of 25 µM chloroquine was analyzed by determining the percentage of Annexin V and propidium iodide (PI) double-positive cells by flow cytometry. (B) EGFR-positive MDA-MB468 (left) and EGFR-negative MDA-MB453 cells (right) were treated with 100 µg/mL of total proteins from supernatants of activated NKL, NKL/GrB-T, or NKL/GrB_S183A_-T cells in the presence of 25 µM chloroquine as indicated. Controls cells were treated with medium containing PMA, ionomycin and chloroquine. After 24 h, the relative number of viable cells was determined in WST-1 assays. (C) To confirm specificity of cell killing, MDA-MB468 cells were pre-incubated with 50 µg/mL of EGFR-specific antibody 425 as a competitor prior to addition of proteins from NK cell supernatants and determination of cytotoxicity as described in (B). Control cells were pre-incubated with isotype-matched control antibody. (D) Dependence of cell killing on GrB activity was confirmed by pre-incubation of culture supernatants from activated NKL cells with 400 µM of GrB-specific peptide aldehyde inhibitor Ac-IETD-CHO before addition to MDA-MB468 cells and determination of cytotoxicity as described in (B). In all cases mean values ± SEM are shown; n = 3 (A); n = 6 (B–D). ***, *P*<0.001; **, *P*<0.01; *, *P*<0.05; ns, *P*>0.05.

Apoptotic cell death is a hallmark of GrB-induced cytotoxicity. To examine whether increased cell death observed upon treatment with GrB-T was due to GrB-induced apoptosis, MDA-MB468 cells were incubated with supernatant from activated NKL/GrB-T cells containing 100 µg/mL of total granular proteins in the presence of chloroquine. Induction of apoptosis was measured by determining the percentage of Annexin V and propidium iodide double-positive cells. After 24 h, 34% of GrB-T-treated cells were apoptotic, while treatment with granular proteins from NKL/GrB_S183A_-T and parental NKL cells both resulted in 20% target cell apoptosis (Fig. 7A).

Next, we investigated whether enhanced cytotoxicity of GrB-T protein was dependent on EGFR expression on target cells. EGFR-negative MDA-MB453 and EGFR-positive MDA-MB468 cells were each treated for 24 h with supernatant from activated NKL/GrB-T cells containing 100 µg/mL of total granular proteins in the presence of chloroquine, and effects on cell viability were determined. As observed before, GrB-T-containing supernatant was highly toxic for MDA-MB468 cells (Fig 7B, left panel), while no significant reduction in viability was observed upon treatment of MDA-MB453 cells (Fig 7B, right panel). To confirm specificity of GrB-T-mediated cell killing, a similar experiment was performed with MDA-MB468 cells pre-treated with EGFR-specific antibody 425 [25]. While in the absence of competitor GrB-T treatment resulted in 54% cell killing, blockade of the ligand binding site of EGFR by the antagonistic antibody abrogated GrB-T binding and reduced cytotoxicity to 30%, which was similar to the levels observed for granular proteins from NKL and NKL/GrB_S183A_-T cells (Fig. 7C).

**Figure 8:**
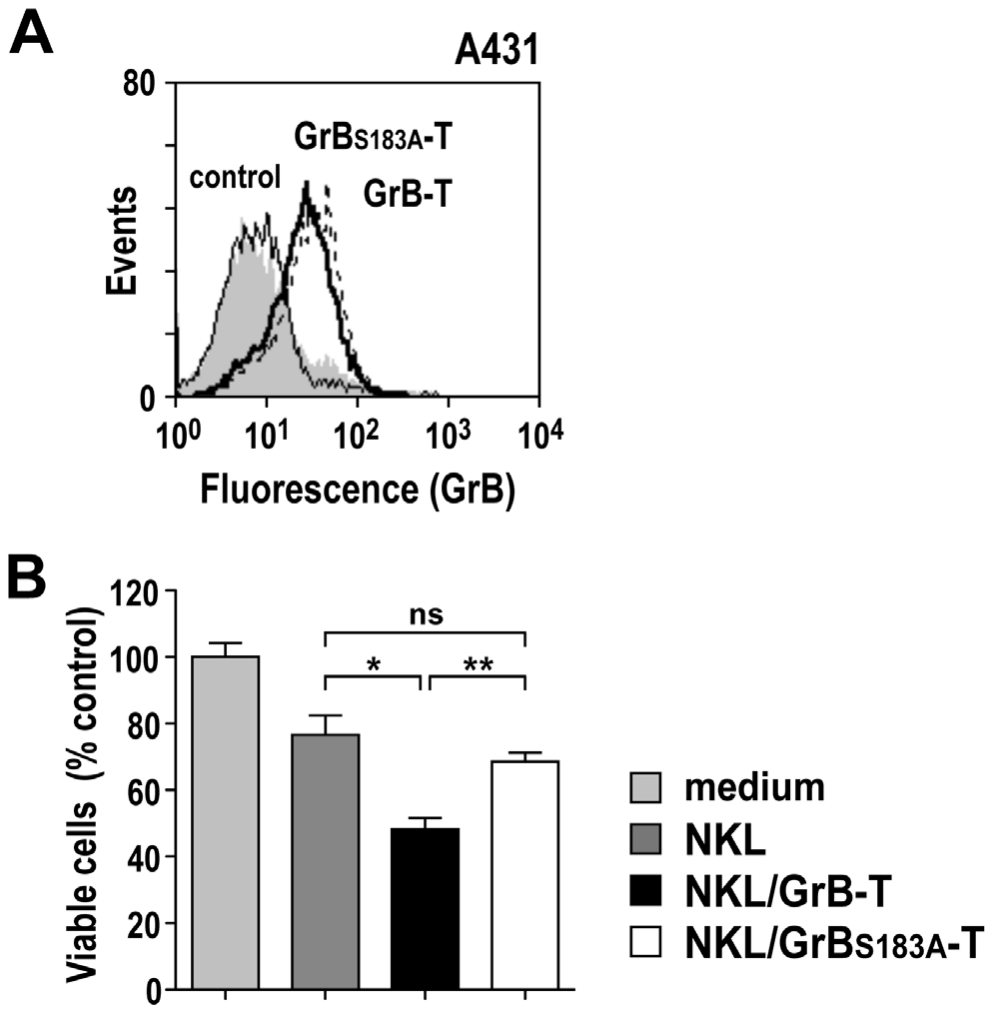
Cell binding and cytotoxicity of GrB-T fusion protein towards A431 cells. (A) Binding of GrB-T (bold line) and mutant GrB_S183A_-T protein (dotted line) released by activated NKL/GrB-T and NKL/GrB_S183A_-T cells to EGFR-positive A431 squamous cell carcinoma cells was determined by flow cytometry with GrB-specific antibody. Cells treated with medium (shaded areas) or proteins released by activated parental NKL cells (regular line) served as controls. (B) To determine cytotoxicity, A431 cells were treated with 100 µg/mL of total proteins from supernatants of activated NKL, NKL/GrB-T, or NKL/GrB_S183A_-T cells in the presence of 100 µM chloroquine as indicated. Controls cells were treated with medium containing PMA, ionomycin and chloroquine. After 24 h, the relative number of viable cells was determined in WST-1 assays. Mean values ± SEM are shown; n = 3. **, *P*<0.01; *, *P*<0.05; ns, *P*>0.05.

The inability of mutant GrB_S183A_-T to enhance cell death indicates that the enzymatic activity of the GrB domain is required for specific cell killing by GrB-T. This was confirmed by pre-treatment of GrB-T from activated NKL/GrB-T cells with the GrB-specific peptide aldehyde inhibitor Ac-IETD-CHO, which markedly reduced specific cytotoxicity (Fig. 7D). Specific binding of GrB-T from NKL/GrB-T cells and high and selective cytotoxicity in the presence of chloroquine was also observed for other EGFR-overexpressing tumor cells such as A431, confirming that these effects are not restricted to MDA-MB468 target cells (Fig. 8).

Taken together, these data demonstrate that GrB-T released by NK cells is bifunctional and selectively cytotoxic for EGFR-overexpressing target cells. Cell killing by GrB-T required TGFα-mediated binding to EGFR on the cell surface, release from vesicular compartments in target cells, and subsequent cleavage of intracellular GrB substrates.

## Discussion

Significant progress has been made over the last decade towards realizing the potential of natural killer cells for cancer immunotherapy [1], [26]. NK cells can respond rapidly to transformed and stressed cells, and have the intrinsic potential to extravasate and reach their targets in almost all body tissues. In experimental models, tumor-specificity of NK cells has been enhanced by genetic modification for expression of chimeric antigen receptors (CAR), that trigger the effector cells' endogenous cytotoxicity upon antibody-mediated recognition of a defined tumor cell surface antigen [27], [28], [29], [30], [31]. Alternatively, antitumoral activity may be augmented by providing NK cells with a targeted effector molecule that is released upon NK cell activation in soluble form, and can act in concert with endogenous natural cytotoxicity mechanisms. As a first step in this direction, here we expressed a chimeric granzyme B fusion protein in human NK cells that carries the peptide ligand TGFα for targeting to EGFR, and evaluated its effect on natural cytotoxicity and its cell killing activity towards EGFR-expressing tumor cells.

We used established NKL cells as a model system [32]. These cells display natural cytotoxicity and can mediate antibody-dependent cell-mediated cytotoxicity (ADCC), but their overall cytotoxic potential is moderate [33]. Transduction with a GrB-T-encoding lentiviral vector resulted in the integration of the provirus into the NKL cells' genome (data not shown), and stable long-term expression of the chimeric fusion protein at levels comparable to endogenous wildtype GrB in these cells. To direct ectopically expressed GrB-T to the lytic granules of the NK cells and facilitate activation-triggered release, we employed complete pre-pro-GrB as an effector domain. Consequently, the chimeric fusion protein like wildtype GrB depends on removal of the signal peptide and subsequent processing by cathepsin C to generate the free N-terminus of mature GrB, that is required for enzymatic activity [7], [14]. Expression and granular storage of GrB-T resulted in a marked increase in total intracellular GrB levels, while not affecting the levels of other granular proteins like perforin.

NKL/GrB-T cells displayed significantly enhanced natural cytotoxicity towards NK-sensitive target cells. This could be attributed to the activity of GrB-T, since NKL/GrB_S183A_-T cells that produce similar amounts of the enzymatically inactive fusion protein only showed cell killing comparable to unmodified parental NKL. Cytotoxicity of NKL/GrB-T cells was abrogated by addition of EGTA, which chelates Ca^2+^ essential for the release of granular proteins and for perforin activity [34], [35], [36]. This suggests that ectopically expressed GrB-T is released via the same mechanism as endogenous wildtype GrB, and likely employs perforin to gain access to GrB substrates in target cells. In contrast to EGFR-negative leukemia and lymphoma cells, EGFR-overexpressing cancer cells of solid tumor origin were resistant to the cytolytic activity of NKL cells. Unexpectedly, this was also the case for NKL/GrB-T cells that express the EGFR-specific fusion protein. This could be explained by insufficient activation of the NK cells upon contact with the NKL-resistant targets. While conjugates were formed between effector and MDA-MB468 target cells, lytic granules were not re-oriented towards the site of contact, and degranulation of NKL cells and release of granular proteins into the immunological synapse was not induced. Hence, GrB-T did not gain access to the target cells.

To facilitate release of GrB-T along with endogenous granular proteins from vesicular compartments, we stimulated genetically modified NKL cells with the phorbol ester PMA and the calcium ionophore ionomycin [37], [38]. Culture supernatant was collected and used for subsequent analysis of the fusion protein. The presence of GrB-T in supernatant and its functionality were confirmed in cell binding experiments and enzymatic assays with a synthetic GrB substrate. We observed strong and selective binding of the fusion protein to EGFR-expressing tumor cells. Despite efficient cell binding, however, GrB-T released from activated NKL/GrB-T cells did not facilitate killing of EGFR-overexpressing tumor cells in the absence of an exogenous endosomolytic activity. Instead, we found the protein trapped in target cells within intracellular vesicles, where it did not have access to its cytosolic substrates.

Under physiological conditions, delivery of wildtype GrB into the cytosol of target cells is aided by the pore-forming protein perforin. This is accomplished either via formation of active perforin pores directly in the cell membrane, or disruption of vesicular membranes after co-endocytosis of GrB and perforin [39], [40], [41]. Enhanced natural cytotoxicity of NKL/GrB-T cells towards EGFR-negative targets suggests that GrB-T like wildtype GrB can cooperate with perforin to enter cells and induce cell death. The TGFα domain contributes approximately 6 kDa to the molecular mass of GrB-T, which is well within the size limit previously suggested for cooperation of GrB fusion proteins or conjugates with perforin [33]. Within the synaptic cleft between fully activated effector cells and targets, high local perforin concentrations can be expected. In contrast, the amount of perforin in culture supernatants of PMA and ionomycin stimulated NKL cells was below the detection limit of immunoblots, and may not have supported cytosolic delivery of GrB-T.

However, when the endosomolytic reagent chloroquine was present, GrB-T from NKL/GrB-T supernatant efficiently killed EGFR-overexpressing tumor cells. Chloroquine is a weak base that accumulates in acidic compartments such as late endosomes and lysosomes, where it interferes with the pH equilibrium, leading to osmotic rupture of the vesicles [42]. It has previously been used to facilitate cytosolic delivery of recombinant GrB fusion proteins and other targeted cell-death inducing proteins in the absence of perforin [15], [20], [43]. Importantly, cell killing activity of GrB-T in the presence of chloroquine was strictly dependent on specific TGFα - EGFR interaction as well as enzymatic activity of the GrB domain. Thereby viability of EGFR-overexpressing breast carcinoma and squamous cell carcinoma cells was reduced to about 50% at a concentration of 100 µg/mL of total granular proteins from activated NKL/GrB-T cells, which corresponds to approximately 5 to 10 ng/mL (125 to 250 pM) of GrB-T as estimated in cytotoxicity assays employing recombinant GrB-T expressed in yeast as a standard [15] (data not shown).

Our data demonstrate that ectopic expression of a targeted GrB fusion protein can enhance antitumoral activity of NK cells. Upon proper activation by target cells, the chimeric GrB-T molecule was released, exhibiting functionality similar to wildtype GrB and markedly enhancing natural cytotoxicity. In addition, the fusion protein displayed EGFR-specific binding and the ability to kill EGFR-expressing tumor cells specifically. Hence, NK cells modified to express a targeted GrB derivative may become therapeutically useful, if NK cell activation at the tumor site and triggered release of the effector molecule can be ensured. This may for example be achieved by co-expressing a targeted GrB fusion protein together with a tumor-specific chimeric antigen receptor of the same or different specificity, that can bypass natural mechanisms preventing activation [31]. Experiments in this direction have been initiated.

## Materials and Methods

### Cells and culture conditions

Human Jurkat acute T cell leukemia cells, C1R-neo B-cell lymphoblastoid cells (both ATCC, Manassas, VA), and human NKL natural killer cells [32] were maintained in RPMI 1640 medium (Lonza, Cologne, Germany). Human MDA-MB453 and MDA-MB468 breast carcinoma cells, A431 squamous cell carcinoma cells, and 293T cells (all ATCC) were cultured in DMEM (Lonza). All media were supplemented with 10% heat-inactivated FBS, 2 mM L-glutamine, 100 U/mL penicillin, 100 µg/mL streptomycin, in addition containing 1.8 mg/mL G418 (C1R-neo), or 10% heat-inactivated horse serum and 200 IU/mL recombinant human IL-2 (Proleukin; Novartis Pharma, Nürnberg, Germany) (NKL).

### Lentiviral GrB-T expression constructs and transduction of NKL cells

cDNA encoding a fusion of human pre-pro-GrB, linked via a flexible peptide linker to human TGFα and C-terminal Myc and His_6_ tags was assembled by stepwise PCR utilizing full-length GrB cDNA [14] and plasmid pPIC9-GrB-T [15] as templates. The GrB-TGFα (GrB-T) sequence was inserted into plasmid pHR'SIN-cPPT-SIEW (pSIEW) [44] upstream of IRES and EGFP sequences of the vector, resulting in the lentiviral transfer plasmid pS-GrB-T-IEW which allows co-expression of GrB-T and EGFP. Following a similar strategy, plasmid pS-GrB_S183A_-T-IEW was generated that encodes the enzymatically inactive control protein GrB_S183A_-T [15]. VSV-G pseudotyped lentiviral vector particles were produced by co-transfecting 293T cells with the respective lentiviral transfer plasmid together with packaging and envelope plasmids pCMVΔR8.91 and pMD2.G [45] using standard calcium phosphate transfection. Culture supernatants containing pseudotyped lentiviral vector particles were collected 48 h later, and sterile filtered to remove cell debris. For transduction, lentiviral particle-containing supernatants were added to NKL cells in the presence of 8 µg/mL polybrene, and the mixtures were centrifuged for 90 min at 32°C and 1,800×g. Then, the cells were cultured overnight at 37°C before replacing the medium. After 48 h, cells were analyzed for EGFP expression by direct flow cytometry using FACSCalibur or FACSCanto II flow cytometers (BD Biosciences, Heidelberg, Germany) and CELLQuest Pro or FACSDiva software (BD Biosciences). Homogeneous pools of EGFP-expressing NKL cells were obtained by two rounds of sorting using a FACSAria fluorescence-activated cell sorter (BD Biosciences).

### Analysis of GrB and perforin expression

Expression of GrB-T and GrB_S183A_-T mRNA was analyzed by semi-quantitative RT-PCR using total RNA from NKL/GrB-T, NKL/GrB_S183A_-T, or parental NKL cells as templates, and oligonucleotide primers 5'-*Sac*II-GrB (5'-CGGCCCGCGGACCATGCAACCAATCCTGCTTCTGCTGGCCTTCC-3′) and 3'-His-*Sac*II (5'-CTACCGCGGCTAGTGATGGTGATGATGGTGATTCAGATCCTC-3'). For immunoblot analysis, NKL/GrB-T, NKL/GrB_S183A_-T, and parental NKL cells were cultured in the presence of GolgiStop^TM^ (BD Biosciences) for 5 h at 37°C. Cells were collected by centrifugation, and lysed by sonication at 4°C in buffer containing 20 mM Tris-HCl, pH 7.5, 150 mM NaCl, 2 mM EDTA, 1% sodium deoxycholate, 1% Triton X-100, 0.25% SDS (all Roth, Karlsruhe, Germany), 1 mM PMSF, 1 mM sodium orthovanadate (Sigma-Aldrich, Taufkirchen, Germany), and protease inhibitor cocktail (Roche Diagnostics, Mannheim, Germany). Subsequently, cleared cell extracts were subjected to SDS-PAGE and immunoblot analysis with GrB-specific antibody 2C5 (Santa Cruz Biotechnology, Heidelberg, Germany), followed by HRP-conjugated secondary antibody and chemiluminescent detection. As a loading control, blots were stripped and reprobed with γ-Tubulin-specific antibody (Sigma-Aldrich).

For intracellular staining, up to 1×10^6^ NKL/GrB-T, NKL/GrB_S183A_-T, or parental NKL cells were cultured in the presence of GolgiStop^TM^ for 5 h at 37°C, collected by centrifugation, washed once with DPBS, and fixed and permeabilized by adding 200 µL of BD Cytofix/Cytoperm^TM^ solution (BD Biosciences) for 30 min at room temperature. Then, cells were washed twice with BD Perm/Wash^TM^ buffer (BD Biosciences), and incubated with either Alexa Fluor 647-conjugated Myc-tag-specific antibody 9E10 (Santa Cruz Biotechnology) for detection of GrB-T and GrB_S183A_-T fusion proteins, Alexa Fluor 647-conjugated human GrB-specific antibody GB11 (BD Biosciences) for analysis of total GrB levels, or phycoerythrin-conjugated perforin-specific antibody δG9 (BD Biosciences) for analysis of total perforin levels. Subsequently, cells were analyzed using a FACSCalibur flow cytometer and CELLQuest Pro software.

### Cytotoxicity assay

Cytotoxic activity of NKL effector cells towards target cells was analyzed in FACS-based assays as described [31]. Briefly, target cells were labeled with calcein violet AM (Molecular Probes, Invitrogen, Karlsruhe, Germany), washed, and then co-cultured with effector cells at various effector to target (E/T) ratios for 4-6 h at 37°C. After co-culture, cells were centrifuged, and 200 µL of a 1 μg/mL propidium iodide (PI) solution were added to each sample shortly before flow cytometric analysis with a FACSCanto II flow cytometer. Specific cytotoxicity was calculated using FACSDiva software. Dead target cells were determined as calcein violet AM and PI double positive. Spontaneous target cell lysis was analyzed in samples containing only labeled target cells, and subtracted to obtain specific cell killing.

### Degranulation of NKL cells

Activation of NKL cells and induction of degranulation was investigated by analyzing surface expression of lysosomal-associated membrane protein LAMP-1 (CD107a) using BD FastImmune^TM^ CD107a APC detection antibody (BD Biosciences) according to the manufacturer's instructions. Briefly, NKL cells (1×10^6^ cells/mL) were either mixed with target cells at an E/T ratio of 1∶1, or stimulated with 1 µg/mL phorbol 12-myristate 13-acetate (PMA) and 1 µg/mL ionomycin (both Sigma-Aldrich) for 60 min at 37°C in the presence of 5 µL of anti-CD107a-APC antibody. Then, 1 µL of GolgiStop^TM^ was added to each sample, followed by incubation for another 4 h at 37°C. Cells were washed, and CD107a expression was analyzed using a FACSCalibur cytometer and CellQuestPro software. To collect proteins released from cytotoxic granules, NKL cells (up to 5×10^6^ cells/mL) were stimulated with 1 µg/mL each of PMA and ionomycin for 6 h at 37°C in serum-free RPMI 1640 medium supplemented with 2 mM L-glutamine, 100 U/mL penicillin, 100 µg/mL streptomycin, and 200 IU/mL IL-2. Culture supernatants were collected and sterile filtered to remove cell debris before subsequent analysis. Yields of total granular proteins from 5×10^6^ cells were typically in the range of 200 to 750 µg.

### GrB activity assay

Enzymatic activity of GrB proteins in supernatants from activated NKL cells was analyzed using the synthetic GrB substrate Ac-IETD-pNA (Alexis, Grünberg, Germany) as described [20]. Cleavage reactions were prepared in duplicates with reaction buffer (10 mM Hepes pH 7.4, 140 mM NaCl, 2.5 mM CaCl_2_) and 200 µM Ac-IETD-pNA in a total volume of 150 µL per sample in 96-well plates. Samples were incubated for 20 h at 37°C, and substrate cleavage was quantified by measuring the absorbance (A) at 405 nm (corrected for background by subtracting A490) with a microplate reader (Molecular Devices, Ismaning, Germany). Peptide substrate incubated in reaction buffer without granular proteins served as blank.

### Binding analysis

To examine EGFR surface expression, target cells (5×10^5^) were incubated with anti-human EGFR antibody R1 (Santa Cruz Biotechnology) for 60 min at 4°C, followed by APC-conjugated goat anti-mouse secondary antibody (Jackson ImmunoResearch, West Grove, PA, USA), and analysis with a FACSCalibur flow cytometer and CELLQuest Pro software. To determine cell binding of GrB-T and GrB_S183A_-T expressed in NK cells, target cells (5×10^5^) were incubated with up to 50 µg of total granular proteins from activated NKL cells for 60 min at 4°C. Unbound proteins were removed, cells were washed, and bound GrB fusion proteins were detected with Alexa Fluor 647-conjugated anti-human GrB antibody GB11 by flow cytometry as described above.

### Cell viability and apoptosis assays

Cytotoxic activity of GrB fusion proteins from NKL cell supernatants was analyzed in WST-1 cell viability assays (Roche Diagnostics) following the manufacturer's recommendations. Target cells were seeded in 96-well plates at a density of 1−2×10^4^ cells/well in triplicates, and incubated for 24 h at 37°C with 50 to 200 µg/mL of total granular proteins from supernatants of activated NKL cells in the presence or absence of 25 µM chloroquine. After addition of WST-1 reagent for 4 h, the relative number of viable cells in comparison to medium-treated control cells was determined by measuring the absorbance (A) at 450 nm (corrected for background by subtracting A690). To compete binding of GrB-T and GrB_S183A_-T to EGFR, target cells were pre-incubated with 50 µg/mL of anti-EGFR antibody 425 [25] or an isotype-matched control antibody for 30 min at 4°C prior to the addition of NKL cell supernatant and analysis of cytotoxicity. Dependence of cytotoxic activity on the enzymatic activity of GrB was confirmed by pre-incubation of total granular proteins from supernatants of activated NKL cells with 400 µM of GrB-specific peptide aldehyde inhibitor Ac-IETD-CHO (Alexis) for 30 min at room temperature before addition to target cells and analysis of cytotoxicity. To determine induction of apoptosis, target cells were seeded in 24-well plates at a density of 2×10^5^ cells/well, and incubated for 24 h at 37°C with 100 µg/mL of total granular proteins from supernatants of activated NKL cells in the presence of 25 µM chloroquine. Control cells were treated with chloroquine-containing medium. For each sample, adherent cells were detached with trypsin-EDTA and combined with cells floating in the culture medium. Harvested cells were washed with PBS, and labeled with Annexin V-APC and PI using the Annexin V Apoptosis Detection Kit (eBiosciences, Frankfurt, Germany) according to the manufacturer's instructions. Fluorescence of cells was measured with a FACSCanto II flow cytometer, and the percentage of apoptotic and dead cells was calculated using FACSDiva software.

### Confocal laser scanning microscopy

To investigate conjugate formation and activation of NKL cells upon target cell recognition, NKL cells were mixed with target cells (E/T ratio of 1∶1), and adhered to glass slides coated with poly-L-lysine (Sigma-Aldrich). After co-incubation for 60 min at 37°C, cells were washed, fixed with 4% paraformaldehyde in PBS for 20 min at room temperature, and permeabilized and blocked for 60 min in buffer containing 0.1% Triton X-100, 3% BSA in PBS. Then cells were incubated with anti-human perforin antibody δG9, followed by Alexa Fluor 546-conjugated anti-mouse IgG (Molecular Probes). Cells were washed twice with PBS, and analyzed with a Leica CTR 6500 laser scanning microscope (Leica Microsystems, Bensheim, Germany). For analysis of uptake of GrB-T fusion protein, MDA-MB468 target cells were grown overnight on coverslips, and then treated for 60 min at 4°C with supernatant of activated NKL/GrB-T or parental NKL cells containing 200 µg/mL of total granular proteins. Then cells were washed, and incubated for another 90 min at 37°C in fresh culture medium to initiate uptake of GrB-T. Cells were fixed with 4% paraformaldehyde in PBS for 20 min at room temperature, permeabilized, and blocked for 60 min in buffer containing 0.1% Triton X-100, 3% BSA in PBS. This was followed by incubation of the cells with 2 µg/mL Alexa Fluor 647-conjugated anti-human GrB antibody GB11 in 3% BSA in PBS for detection of GrB-T. Nucleic acids were counterstained with DAPI. Cells were washed twice with PBS, and analyzed with a Leica CTR 6500 laser scanning microscope.

### Statistical analysis

Differences between values were evaluated using the two-tailed unpaired Student's *t* test. *P* values <0.05 were considered significant. Statistical calculations were done using Prism 5 software (GraphPad Software, La Jolla, CA).
